# Presumptive risk factors for monkeypox in rural communities in the Democratic Republic of the Congo

**DOI:** 10.1371/journal.pone.0168664

**Published:** 2017-02-13

**Authors:** Claire A. Quiner, Cynthia Moses, Benjamin P. Monroe, Yoshinori Nakazawa, Jeffrey B. Doty, Christine M. Hughes, Andrea M. McCollum, Saturnin Ibata, Jean Malekani, Emile Okitolonda, Darin S. Carroll, Mary G. Reynolds

**Affiliations:** 1 Division of Epidemiology and Biostatistics, School of Public Health, University of California, Berkeley, California, United States of America; 2 US Centers for Disease Control and Prevention, Poxvirus and Rabies Branch, Atlanta, Georgia, United States of America; 3 International Conservation Education Fund, Washington, D.C., United States of America; 4 University of Kinshasa, Department of Biology, Kinshasa, Democratic Republic of Congo; 5 Kinshasa School of Public Health, Kinshasa, Democratic Republic of Congo; University of Florida, UNITED STATES

## Abstract

*Monkeypox virus* (MPXV), a close relative of *Variola virus*, is a zoonotic virus with an unknown reservoir. Interaction with infected wildlife, bites from peri-domestic animals, and bushmeat hunting are hypothesized routes of infection from wildlife to humans. Using a Risk Questionnaire, performed in monkeypox-affected areas of rural Democratic Republic of the Congo, we describe the lifestyles and demographics associated with presumptive risk factors for MPXV infection. We generated two indices to assess risk: Household Materials Index (HMI), a proxy for socioeconomic status of households and Risk Activity Index (RAI), which describes presumptive risk for animal-to-human transmission of MPXV. Based on participant self-reported activity patterns, we found that people in this population are more likely to visit the forest than a market to fulfill material needs, and that the reported occupation is limited in describing behavior of individuals may participate. Being bitten by rodents in the home was commonly reported, and this was significantly associated with a low HMI. The highest scoring RAI sub-groups were ‘hunters’ and males aged ≥ 18 years; however, several activities involving MPXV-implicated animals were distributed across all sub-groups. The current analysis may be useful in identifying at-risk groups and help to direct education, outreach and prevention efforts more efficiently.

## Introduction

The virus that causes human monkeypox (MPX), *Monkeypox virus* (MPXV) is a close relative of *Variola virus* (> 90% genome homology),[1][2] the causative agent of smallpox, and is distinguished by the broad range of animal taxa which it can infect, including humans, rodents and non-human primates. Symptoms of MPX closely resemble those of smallpox, and include a febrile prodrome followed by development of a disseminated vesiculo-pustular rash. [3] MPX patients are vulnerable to secondary bacterial infection, dehydration, encephalitis, bronchopneumonia and blindness due to corneal scarring from lesions. [4]^,^ [5]

Human MPX cases have been reported in multiple countries of Central and West Africa. [6] The majority of reports of disease are from the Democratic Republic of the Congo (DRC). Recent reports have found the annual crude incidence of human MPX to be 5.53 per 10,000 people, in endemic regions of DRC where most of the infected are under the age of 15 years. [7] Humans acquire MPXV from either contact with an infected animal (primary zoonotic transmission) or from stuttering chains (R_0_ <1) propagated by inter-human transmission. [8] To date, the virus has only been isolated twice from wild animals, once from a squirrel (*Funisciurus sp*.) in DRC and once from a sooty mangabey (*Cercocebus sp*.).[9]^,^ Hunting and butchering of bushmeat are presumed to be risk activities for primary zoonotic transmission. [10] Hunting bushmeat is a common traditional and commercial practice in Central Africa and is the presumed mechanism for how several relevant human pathogens have entered human populations, including Ebola virus and human immunodeficiency virus (HIV).[11]^,^[12] Studies suggest that contact with bodily fluids, i.e. blood, salivary/respiratory droplets, lesion exudate and crust [13] from an infected human are involved in the transmission of the disease. Additionally, experimental disease transmission animal studies using the prairie dog model, which was shown to imitate key characteristics of human MPX disease, have shown that MPXV could be transmitted from one prairie dog to another naïve prairie dog via fomites. [1] Thus, caretakers and housemates of infected individuals are likely at risk for inter-human transmission.

Here we evaluate data from a Risk Questionnaire, conducted by the International Conservation and Education Fund (INCEF) and report common practices of residents in at-risk villages in the Congo Basin, prevalence of presumed risk factors for MPXV infections and transmission (for zoonotic and inter-human transmission), and proxies for risk-associated behaviors. The goals of this work included describing common behaviors and practices surrounding fulfilling nutritional requirements in this population, quantifying risk behaviors, identifying sub-groups who are most likely to partake in those behaviors, and external indicators that may explain or predict risk-associated behaviors. Further, based on these analyses, we propose hypotheses about MPXV trends in these communities.

## Methods

A Risk Questionnaire was designed by INCEF in partnership with local community members and with technical input provided by US Centers for Disease Control and Prevention. The questionnaire was created to capture and better understand presumptive risk factors for MPXV infection and the population that is most likely to participate in these activities, with the objective of improving the specificity and efficacy of outreach materials for populations in rural DRC. This survey was performed in Tshuapa Province, DRC, a rural, forested area of the Congo Basin, in which MPX is endemic. To our knowledge, this questionnaire is the first of its kind, to be conducted in this population by the investigators.

The questionnaire consisted of a series of, multiple choice, and yes/no questions addressing the current practices in these communities, which are presumed to lead to MPXV infection such as hunting of animals indicated as potential reservoirs of the virus as well as other behavioral and demographic descriptors: (i.e., types and number of domestic animals owned, types of animals that they had hunted in the last month, number of people living in a given household, age, sex, occupation, and if they themselves or housemates had been bitten by a rodent in their home). Respondents, in addition to the questions addressing presumptive risk factors, were also asked their weekly frequency of visiting common locales—market, church, forest and school. Two slightly different versions of the questionnaire were used, one in 2011/2012 and one in 2013. Modifications were made to reduce redundancy in questions, as well as to collect more information about activities that were common in the forest, specific materials used for housing structures, and types of interaction participants had with specific animal species in the one month prior (photos were used to aid in the accurate identification of species).

A team of two facilitators, one versed in health education and the other an experienced community outreach coordinator, administered the questionnaire across 38 villages. Written permission from local government officials was obtained prior to the mission. Additionally, upon arrival in each a village, facilitators met with village leaders to obtain verbal permission to perform the work. After being dividing participants into sub-groups by age and sex, facilitators described the Risk Questionnaire, its purpose, and voluntary nature of the process. Verbal consent was obtained from participants and, for participants younger than 18 y/o, consent from their parents/guardians was obtained. Participants were then asked to volunteer themselves, by raising hands, for questionnaire participation. Each volunteer was questioned individually in a private setting. The questionnaires were delivered in the local language, Lingala, and recorded in French by facilitators who fluently speak and routinely use both languages. A total of 939 participants were surveyed-589 in 2011/2012 and 350 in 2013.

### Ethics statement

The survey was determined to not be research by the Centers for Disease Control and Prevention. Prior to requesting volunteers, the evaluation methods, purpose, and voluntary nature of the survey was described to prospective participants. Verbal consent was obtained from participants or the parents/guardians of those younger than 18 y/o.

## Analysis

Individual and household level variables were generated and provided information about (i) the incidence of MPXV risk factors in these communities, (ii) the sub-groups that were at higher risk, and (iii) two proxies that could be used to predict behavior. A series of comparisons were performed to determine which sub-groups and characteristics were associated with the presumptive risk factors for MPXV.

An age categorization was generated to distinguish school, intermediate and mature aged participants: ≤ 17 years old (y/o), 18–35, and #x2265;36. Data concerning the frequency in which a participant attended various destinations were categorized according to the frequency of visits per week as follows: church/mosque, 0, 1–3, 4–5; forest, 0, 1–2, 3–4, #x2265; 5; market, 0, 1–2, #x2265; 3; school, 0, 1–5, #x2265; 6. A household size variable was generated as follows: 1–4 people per household, 5–10, #x2265; 11. The number of different animal species owned by a given household was generated as follows: 0, 1–2, 3 or more.

Data about the materials used for housing structures were summarized in the Household Materials Index (HMI). A value of 0 or 1 was assigned to each material of the four components that make up a house (floor, walls, roof and door). Materials that were available at no presumed economic cost such as dirt, tiger palm and thatch, were scored with a 0 and materials that had a presumed cost such as brick, cement, and metal were assigned a 1. The value for each of the four housing components were summed, such that each household earned a single HMI score ranging from 0 to 4. For example, a household with a dirt floor (0), a thatch door (0), a metal roof (1) and brick walls (1), scored an HMI of 2. Very few households scored HMIs of 3 or 4; thus categories 2–4 were collapsed into a single category resulting in three HMI levels were created, 0 = poor, 1 = intermediate and 2–4 = good.

For the purposes of this analysis, MPXV implicated animals were separated into three categories: non- human primates, [14,15] rodents (other than squirrels) [10,16] and squirrels. [9,17] Squirrels formed their own category due to their potential role in virus transmission to humans. [9]^,^[17] A Risk Activity Index (RAI) was created by summing the number of interactions that an individual reported having engaged in with MPX-implicated and other animals, within the last month. For example, an individual who reported killing two squirrels, butchering three rodents and eating one non-human primate in the last month, scored an RAI of six. Individuals scoring 0 were considered to have low risk. Participants who only reported interactions with animals not implicated in MPXV infection (including genets, goats, and pigs) were scored as medium risk, as time spent in the forest and with material from the forest, could lead to infection via body fluids (i.e. excrement, saliva, blood, etc.) of infected animals, as well. Participants who reported 1–4 interactions with MPX-implicated animals were scored as high risk, and #x2265; 5, as elevated risk. HMI and RAI could only calculated for participants interviewed in 2013 because necessary data was not collected in 2012.

Details regarding the zoonotic transmission of MPXV are not yet defined; therefore, we were limited to exploring presumptive risk factors rather than defined risk categories. For this analysis, the presumptive risk factors used were HMI, number of species owned, household size, report of being bitten by rodents, type of contact with MPXV-implicated animals, and RAI. These presumptive risk factors were compared across age, sex and occupation. Odds ratios, with corresponding 95% confidence intervals, and chi square test p-values for these comparisons were calculated. Where indicated, the Fisher’s Exact test was used. Significance was determined as p<0.05. SAS v. 9.3, statistical software was used for all statistical analyses.

## Results

### Population characteristics

A total of 939 participants were surveyed, 64% male. The largest age category (n = 387, 41%), was ≥36 y/o. The most common reported occupation was ‘farmer’ (n = 337, 35.9%), followed by “student” (n = 219, 23.3%) (Table 1).

**Table 1:** Demographic data from survey respondents.

Variable	% (n)
**Sex**	
**Male**	64% (598)
**Female**	36% (331)
**Age**	
**≤17**	28.0% (263)
**18–35**	30.8% (289)
**≥36**	41.2% (387)
**Occupation**	
**Farmer**	35.9% (337)
**Student**	23.3% (219)
**Other**	15.2% (143)
**Hunter**	11.5% (108)
**Housewife/ employee**	8.5% (80)
**Child not attending school**	5.2% (49)
**Vendor**	1.1% (10)

**Fig 1:**
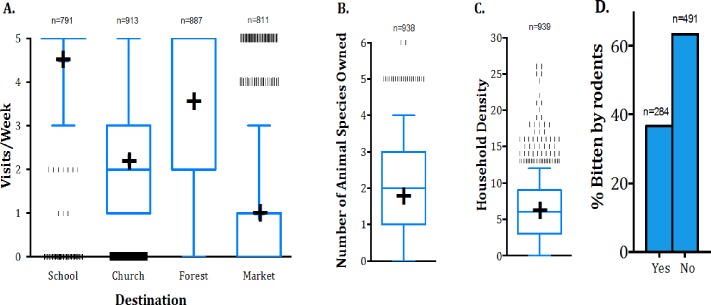
**A-C: Reported activities, ownership of animals, household density, and rodent bites of respondents.** The blue plus sign (+) represents the median, the top and bottom of the box represent the 25^th^ and 75^th^ percentiles, respectively, and the whiskers represent the 10th and 90^th^ percentile in each data set. Data points below and above the 10^th^ and 90^th^ percentile are drawn as vertical lines (**|**). **A**—Reported frequency of visits to church, forest and market and reported frequency of children in household attending school, weekly. **B**—The number of distinct animal species owned by a given household. **C**—Distribution of number of people living in a given household. **D**—Percent of people who reported having been bitten by rodents in their household.

Most households reported sending children to school at least 5 days/week (n = 739, 92.1%) while less than 10% reported never sending children to school (n = 63, 7.9%). Most participants attended a church/mosque more than once/week (n = 538, 58.74%) while less than 10% reported never visiting religious buildings (n = 72, 7.86%). Multiple visits to the forest were very common among participants; half reported going five or more times/week (n = 457, 50.72%) and nearly 90% reported going at least once/week, (n = 803, 89.13%). To the contrary visits to the market were less common; more than half reported never going to the market, (n = 443, 53.63%) and only a small proportion reported going more than twice/week, (n = 120, 14.53%) (Fig 1A).

Regarding activities performed during visits to the forest (data only collected in the 2013 version of survey), the majority of respondents reported agriculture activities, (n = 231, 84.93%). Approximately half of the respondents reported collecting firewood (n = 158, 58.30%), water (n = 144, 53.73%), and foraging (n = 126, 46.49%). Interestingly, more than 20% reported hunting in the forest (n = 62, 22.88%). The number of people who reported hunting was almost twice the number of people who identified their occupation to be ‘hunter’; and the number of people who reported farming was almost three times the number of people who identified their occupation to be ‘farmer’ (Table 2).

**Table 2:** Comparison of Occupation to Reported Activities.

Reported Occupation	Percentage (n = 939)	Reported Activity^£^	Percentage (n = 270)
Hunter	11.5% (108)	Hunts	23.0% (62)
Farmer	35.9% (337)	Farms	82% (231)

^**£**^ = “What activity do you do in the forest?” was a question included only in the 2013, modified version of the survey, thus the change in denominator.

Animals most commonly owned by the surveyed population were chickens (n = 541, 57.6%), ducks (n = 367, 39.13%), goats (n = 367, 31.13%), dogs (n = 154, 16.42%) and pigs (n = 140, 14.93%). Participants reported owning a range of 0–6 distinct animal species, (mean = 1.8, SD = 1.3), and most owned less than three animal species (n = 667, 71.11%) while 19% did not own any animals (n = 182, 19.4%) (Fig 1B). More than 30% of participants reported having been bitten by rodents in their household (n = 284, 36.65%), (Fig 1D).

The household size of the participants ranged from 1–26 people (mean 7.6, SD 3.9). (Fig 1C). A positive association was found with household size and the number of animal species owned (*χ*^2^ p-value = 0.0005, df = 4). Additionally, ‘hunters’ were significantly more likely to live in the largest household size (11+ people) relative to the smallest size household (less than 5 people) (OR = 2.15, CI = 1.24–3.70).

### Household Materials Index

The distribution of HMI levels is presented in Table 3.

**Table 3:** Proportion of respondents by Household Materials Index (HMI) and Risk Activity Index (RAI) levels.

HMI	Percentage (n)
**Poor**	14.7% (49)
**Intermediate**	31.4% (105)
**Good**	53.9% (180)
**RAI**	
**Low**	13.1% (46)
**Medium**	8.8% (30)
**High**	47.7% (167)
Elevated	30.6% (107)

**Fig 2:**
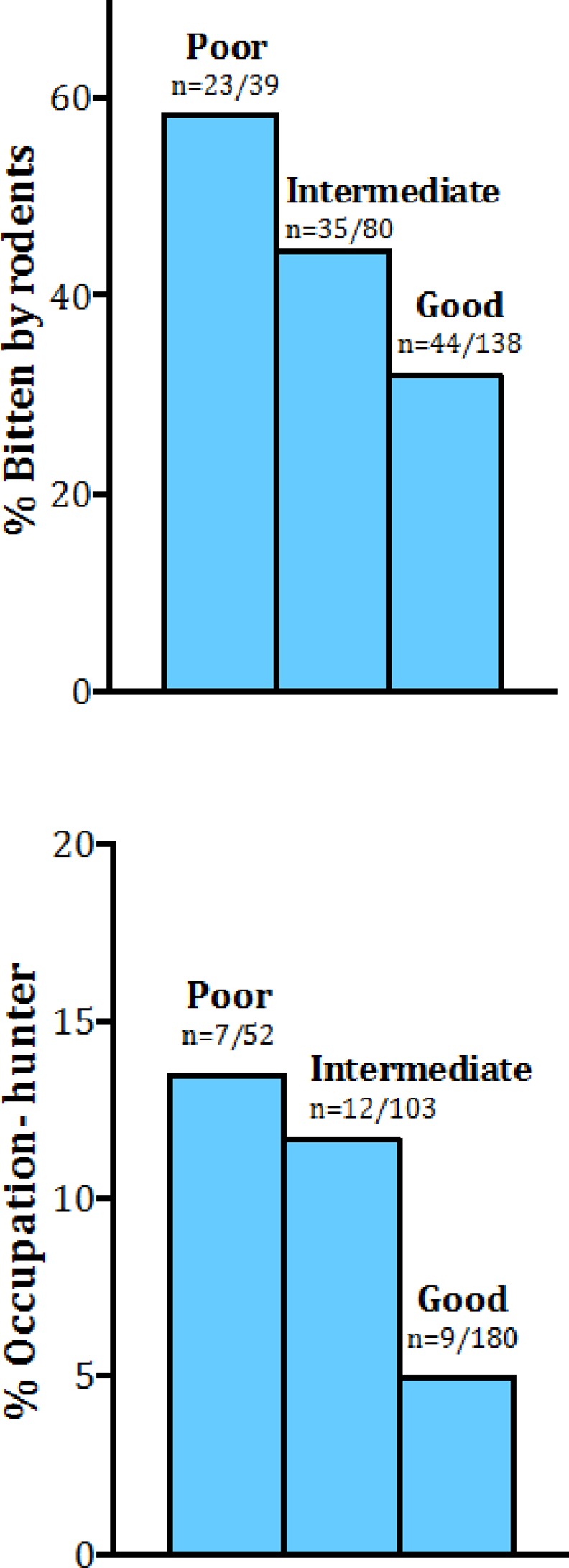
**A-B: Associations of Household Materials Index (HMI) and presumptive monkeypox (MPX) risk factors. A.** reported themselves or others as having been bitten by rodents in their household (n = 102), *χ*^2^ p value = 0.0084, **B.** reported their occupation as a hunter (n = 28) *χ*^2^ p value = 0.054.

There was a negative association between HMI (data only collected in the 2013 version of survey) and reported history of rodent bites within the household, (*χ*^22^ p = 0.002, df = 2), (Fig 2A), with those in the poor HMI category nearly three times as likely to have reported rodents bites than those who scored a good HMI, (OR = 2.99, CI = 1.4–6.35). There was also a marginally significant association between HMI level and reported occupation of hunter vs occupation of “not-hunter” (*χ*^2^ p-value = 0.054, df = 2), (Fig 2B). Individuals with a poor HMI were more than twice as likely to report “hunter” as their occupation as compared to those who had a good HMI, (OR = 2.65, CI = 0.89–7.69).

### Interactions with wild animals

Sex, age, and occupation were found to be associated with some behaviors that placed the respondents in contact with MPX-implicated wildlife. Males are more than eight times as likely to have hunted at least one non-human primate in the previous three months than females (OR = 8.45, CI = 1.98–36.07) (Table 4).

**Table 4:** Associations of presumptive risk factors for MPXV infection and demographics.

		Age (reference = ≤17 y/o)					Gender (reference = female)		
		18–35 y/o		#x2265;36 y/o		*χ*^2*2*^ *p-value (2 df)*^*†*^	Male		*Χ*^*2*^ *p-value (1 df)*^*†*^
		OR	(95% CI)	OR	(95% CI)		OR	(95% CI)	
**Non-human primates**	**Hunt**	4.66	(0.60–37.5)	6.04	(0.79–46.0)	*0*.*141*	**8.45**	**(1.98–36.1)**	*<0*.0001*
	**Find**	0.60	(0.25–1.44)	0.71	(0.32–1.58)	*0*.*514*	1.58	(0.82–3.04)	*0*.210*
	**Eat**	1.11	(0.52–2.34)	0.67	(0.34–1.34)	*0*.*130*	0.97	(0.60–1.56)	*1*.00*
	**Dismember**	1.31	(0.66–2.61)	0.87	(0.46–1.70)	*0*.*257*	0.89	(0.57–1.41)	*0*.650*
	**Sell**	3.60	(0.79–16.40)	2.04	(0.45–9.23)	*0*.*126*	1.29	(0.58–2.91)	*0*.690*
**Rodent (non- squirrel)**	**Hunt**	0.70	(0.24–2.03)	0.90	(0.34–2.37)	*0*.*752*	**6.26**	**(1.87–20.9)**	*<0*.0001*
	**Find**	0.76	(0.24–2.40)	0.76	(0.26–2.21)	*0*.*871*	0.83	(0.38–1.83)	*0*.684*
	**Eat**	1.27	(0.59–2.74)	1.09	(0.52–2.25)	*0*.*772*	1.44	(0.86–2.43)	*0*.200*
	**Dismember**	0.92	(0.42–2.02)	0.97	(0.47–2.02)	*0*.*974*	1.31	(0.76–2.24)	*0*.358*
	**Sell**	2.22	(0.10–47.0)	2.97	(0.16–54.6)	*0*.*485*	1.30	(0.25–6.81)	*1*.00*
**Squirrel**	**Hunt**	N/A		N/A		N/A	N/A^‡^		
	**Find**	1.09	(0.33–3.67)	0.70	(0.21–2.30)	*0*.*593*	1.11	(0.46–2.65)	*1*.00*
	**Eat**	0.55	(0.27–1.10)	0.33	(0.17–0.64)	***0*.*003***	0.88	(0.53–1.45)	*0*.610*
	**Dismember**	0.46	(0.22–0.93)	0.30	(0.15–0.59)	***0*.*002***	0.93	(0.53–1.57)	*0*.790*
	**Sell**	7.98	(0.45–141.0)	N/A		*0*.002 (1 df)*	3.71	(0.45–30.5)	*0*.273*

OR = Odds Ratio

CI = Confidence interval

† = Chi-square p-value (degrees of freedom), unless otherwise noted. Statistically significant values are bolded

* = Fisher's Exact test p-value (degrees of freedom), statistically significant values are bolded

‡ = No participants in this category answered affirmatively

The four largest occupation groups were also tested. Hunters were 60 times more likely (OR = 60.5, CI = 6.8–535.0) and farmers were twice as likely (OR = 2.3, CI = 0.28–18.9) to have hunted this animal group compared to students (Table 5).

**Table 5:** Associations of presumptive risk factors for MPXV infection and occupation.

		Occupation (reference = "Student")						
		Hunter		Farmer		Housewife/Employee		*Χ*^*2*^ *p-value (3 df)*^*†*^
		OR	(95% CI)	OR	(95% CI)	OR	(95% CI)	
**Non-human primates**	**Hunt**	60.50	(6.83–535.9)	2.30	(0.28–18.9)	0.96	(0.04–24.7)	***<0*.*0001***
	**Find**	1.07	(0.28–4.0)	0.81	(0.35–1.87)	0.12	(0.01–2.3)	*0*.*305*
	**Eat**	2.67	(0.67–10.6)	0.96	(0.48–1.94)	0.33	(0.10–1.1)	*0*.*062*
	**Dismember**	2.68	(0.83–8.69)	1.41	(0.73–2.74)	0.48	(0.14–1.6)	*0*.*083*
	**Sell**	39.60	(4.49–349.3)	3.87	(0.49–30.4)	3.14	(0.18–53.6)	***<0*.*0001***
**Rodent (non- squirrel)**	**Hunt**	1.73	(0.43–7.01)	0.77	(0.28–2.08)	0.20	(0.01–3.69)	*0*.*261*
	**Find**	0.94	(0.17–5.34)	0.82	(0.28–2.40)	0.57	(0.06–5.32)	*0*.*960*
	**Eat**	1.60	(0.51–5.03)	1.00	(0.47–2.12)	1.38	(0.39–4.85)	*0*.*772*
	**Dismember**	1.43	(0,44–4.65)	0.96	(0.44–2.06)	1.55	(0.43–5.51)	*0*.*760*
	**Sell**	N/A^‡^		2.01	(0.10–39.6)	N/A^‡^		*0*.*684 (2 df)*
**Squirrel**	**Hunt**	N/A^‡^		N/A^‡^		N/A^‡^		N/A^‡^
	**Find**	1.21	(0.20–7.22)	0.83	(0.25–2.70)	0.30	(0.02–5.85)	*0*.*661*
	**Eat**	0.69	(0.22–2.16)	0.48	(0.24–0.96)	0.38	(0.09–1.52)	*0*.*174*
	**Dismember**	0.59	(0.18–1.93)	0.43	(0.21–0.87)	0.41	(0.10–1.67)	*0*.*116*
	**Sell**	7.38	(0.26–189.5)	3.80	(0.21–68.8)	N/A^‡^		*0*.*467 (2 df)*

OR = Odds Ratio

CI = confidence interval

† = Chi-square p-value (degrees of freedom), unless otherwise noted. Statistically significant values are bolded

* = Fisher's Exact test p-value (degrees of freedom), statistically significant values are bolded

‡ = No participants in this category answered affirmatively

All occupational groups were more likely to have sold a non-human primate than students: hunters, 39 times more likely (OR = 39.6, CI = 4.49–349.30), farmers (OR = 3.87, CI = 0.49–30.37), and housewives/employees (OR = 3.14, CI = 0.18–53.59) were each more than three times as likely than students. Similar patterns were found in this population among those who hunt rodents; males were more than six times as likely as females (OR = 6.3, CI = 1.87–20.90) to have reported hunting rodents. While not significant, students were more than five times as likely to have reported hunting rodents as housewives/employees (OR = 5.10, CI = 0.27–100.0).

A different pattern was observed among those who had reported interactions with squirrels. Significant associations were found between age and eating (p value = 0.003), dismembering (p value = 0.002), and selling (p value = 0.002) squirrels (Table 4). The older age groups (18–35 and #x2265;35 y/o) were significantly less likely to have reported dismembering a squirrel when compared to the youngest age group (≥ 17 y/o) (OR = 2.17 CI = 1.08–4.55 and OR = 3.33 CI = 1.69–6.66, respectively). Those >35 y/o were also significantly less likely to have reported eating a squirrel than those in the youngest age group (≥ 17 y/o) (OR = 3.03 CI = 1.56–5.88). While not statistically significant, those who were most likely to report selling squirrels were those in the 18–35 y/o age group (OR = 7.98, CI = 0.45–141.0) (Table 4).

### Risk Activity Index

Forty-six participants had a low RAI score (data only collected in the 2013 version of survey), 30 medium, 167 high and 107 elevated (Table 3). Sex was significantly associated with RAI (*χ*^2^ p-value = 0.023, df = 3) whereby males were more likely to score the riskiest RAI levels than females. Occupation was associated with RAI (*χ*^2^ p-value = 0.0013, df = 9). For example, hunters were more likely to be categorized into the elevated or high-risk RAI levels compared to other groups (or compared to student reference group). There was a significant inverse association between the frequency of church attendance and the RAI score (*χ*^2^ p-value = 0.0083, df = 9), in which those who reported a higher frequency of church attendance were more likely to have a lower RAI score than those who attend less frequently or not at all.

**Fig 3:**
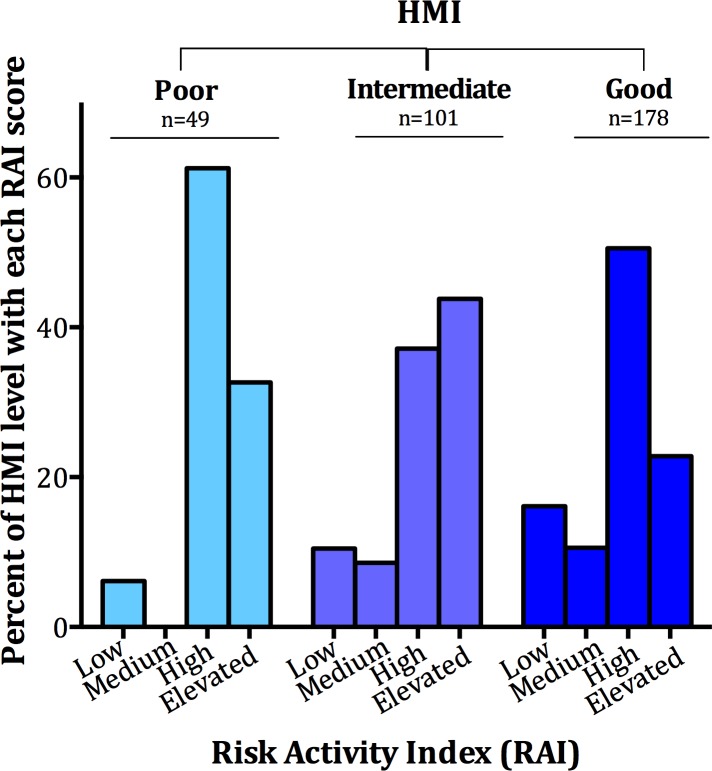
Association of Household Materials Index (HMI) and Risk Activity Index (RAI). Plotted is the percentage of each HMI level that ranks at each level of the RAI (*χ*^2^ p value = 0.0008. df = 6).

Finally, we compared HMI and RAI and a significant, inverse association was found (*χ*^2^ p value = 0.008, df = 6); where lower risk of presumptive MPX activities was found among individuals who scored an intermediate and good HMI (Fig 3). Of those in the poorest HMI category, 61.2%, (n = 30) scored a good RAI score and, 32.7% of them (n = 16) scored an elevated RAI. As a comparison, individuals scoring the highest HMI level, had a smaller proportion scoring as high risk RAI, (50.6%, n = 91), and also a smaller proportion of this group scored an elevated RAI (n = 41, 22.8%). Of those who scored a good HMI, very few scored a low risk RAI (16.1%, n = 29) or medium risk (10.6%, n = 19). This suggests that HMI, demonstrated here as a socioeconomic indicator, is associated with MPX risk behavior.

## Discussion

This study was performed to describe the presumptive risk factors for human MPXV infection in communities at risk for sylvatic zoonoses. A disease outcome (i.e. a diagnostic to determine if a participant had previously been exposed to MPXV) was not available in this study. As such is the goal with studies of many emerging infectious diseases, an aim here was to better inform future research. Accordingly, the information included herein, allows us to make predictions about who in these communities is at highest risk for MPXV infection, to help understand societal norms of behavior and practices which could lead to disease transmission, and to identify indicators of poverty which may be indicative of practices that lead to disease acquisition and/or transmission within households. The hypotheses generated from this work are not only applicable to MPX but may be relevant to other zoonotic diseases associated with sylvatic animals in this region as well, such as Ebola or Marburg viruses.

We examined the patterns of human behavior and social activity in these populations. The community examined consisted of a population that is forest-dependent and largely fulfills material and nutritional needs via forest resources rather than purchases at a market. Many villages are surrounded by forest and they do not have markets of their own; thus, visiting a market can involve significant travel. Frequent (multiple times/week) activities included school and church attendance.

There were certain occupational, gender and age groups that were more likely to participate in activities with MPXV-implicated animals than others. Males were more likely than females to report hunting of both non-human primate and rodent animal groups. However, age was the best predictor of those who interacted with squirrels. The youngest age group, ≥ 17 y/o, was the most likely to have reported eating and dismembering squirrels, relative to the other age groups, 18–35 and #x2265; 35 y/o. It was found that those who identified their occupation as ‘hunter’ were the most likely to have reported hunting and selling a non-human primate, it is noteworthy, that ‘farmers’ also reported having done so. The occupational groups of housewives/employees and farmers were more than three times as likely as the reference group to have reported selling non-human primates.

These findings suggest that some occupational groups are at a higher risk for primary zoonotic MPXV infection than others. However, at least one behavior, involving an interaction with MPX-implicated animals was reported by three of the four occupational groups–hunters, farmers and housewife/employee and by each of the three age groups. This is consistent with the assertion that occupation has limited utility in describing behavior. These finding highlight the need for an alternate means to identify risk for zoonotic disease transmission in these communities. Here, we show the utility of one such tool, the RAI, by analyzing its association with high-risk groups: males, hunters, and those living in low HMI households.

While behavior will remain an area of focus in prevention of MPXV infection, other factors may also contribute to MPX risk, such as household size. We considered the hypothesis that risk of primary MPXV infection be associated with larger households, which could be explained by the higher protein requirements that a larger household would have, thus, potentially increasing dependence on and contact with wild animals as a source of meat. The result that ‘hunters’ were more likely to live in higher density households supports this hypothesis.

Another tool to identify vulnerability is that of HMI, which was designed in this study as a proxy for socio-economic status (SES). SES is an established indicator for a myriad of infections, including HIV/AIDS, plasmodium, helminthes and tuberculosis. [18]^,^[19]^,^[20]. The HMI is indicative of the quality of a housing structure, which we predicted would directly impact household permeability to peri-domestic rodents to enter the household and then defecate, urinate or bite humans inside. Indeed, the data showed that being bitten by rodents in the home was found to be positively associated with a low HMI. Rodent traps placed in domiciles in this region of the DRC have resulted in the capture of the following species: shrews of the genus *Crocidura*, black rats (*Rattus rattus*), roof rats (*Rattus norvegicus*), house mice (*Mus spp*.), dormice (*Graphiurus sp*.), and brush-furred mice (*Lophuromys spp*.). Two other species of rodents have been caught near houses: African wood mice (*Hylomyscus stella*) and rusty-nosed rats (*Oenomys spp*) (J. Doty, Personal communication). Of these rodents, only dormice have been previously implicated in MPXV infection and transmission. [21] Although there is not strong evidence that rodent bites in the home are a significant feature of MPXV transmission in areas of endemic disease, this feature of human-animal interaction warrants further investigation.

Finally, there was an inverse relationship between risk of MPXV acquisition and SES as demonstrated by the comparison of HMI and RAI. An interpretation of this relationship is that higher SES households are able fulfill protein requirements through alternate means and depend less on bushmeat as a source for protein. This results in fewer interactions with and potential exposures to MPXV implicated reservoirs.

While the importance of culture and tradition in these communities should be a major consideration for any prevention or intervention efforts, these data suggest that in this population, bushmeat hunting and consumption may be motivated as much by necessity as by tradition. It will be critical to understand the relative influence of each in order to devise effective disease prevention messages and programs. This would also extend to other zoonotic infections as well, such as Ebola virus. As was observed in the 2014 Ebola epidemic in West Africa, bushmeat consumption/interaction can have far-reaching, even transnational, impacts. [22] The same region of the DRC where the present study was conducted also experienced Ebola outbreaks as recently as 2014. [23] Where humans hunt and consume sylvatic protein, the expectation of and vigilance for zoonotic diseases in humans should be maintained.

Here, we described populations within the Congo Basin of DRC that may have higher risk for contracting MPX, either through inter-human or zoonotic transmission routes. The number of primary zoonotic transmission cases is likely to be a function of the frequency and type of interactions that individuals have with infected wildlife. However, circumstances that are apt to be associated with primary zoonotic introductions may also be associated with increased opportunities for inter-human transmission (i.e., hunters tending to be part of large households). This makes the household context an important indicator of risk for disease, as has been observed with other communicable diseases. [24,25] Based on these findings, we can formulate the following hypotheses for primary zoonotic and inter-human transmission cases of MPX:

- Primary zoonotic MPXV infection is more likely to occur in males than females.
- Males, aged #x2265; 17, with the occupation, ‘hunter’ are the most likely to be the primary zoonotic MPXV (debut) case in a household.
- The incidence of primary zoonotic MPX increases with a higher RAI.
- Households scoring a low HMI will experience more primary transmission of MPXV than households with higher HMI.
- Households that have a higher risk of primary zoonotic MPXV infection will also have a higher risk of inter-human transmission.
- The incidence of inter-human transmission of MPXV increases in parallel with the number of persons per household.

These hypotheses could be used to better direct future research as well as educational and prevention efforts for MPX. The indices that were created (HMI and RAI) and indicators for risk (occupation, sex, age, household density, report of being bitten by rodents) that are shown to correlate with presumptive risk factors, could be used to easily identify high-risk individuals within similar communities.

We acknowledge the limitations of this work. A disease or exposure outcome was not available in this study. Accordingly, the information included herein, allows us to make predictions about who in these communities is at highest risk and societal norms of behavior, which may be indicative of practices that lead to zoonotic disease acquisition and/or transmission in a community. Additionally, data were subjected to reporting bias in that the sample population may not have been expressly representative of underlying populations, as evidenced by the skewed age distribution and sex bias. Accurate census data were not available to allow us to correct for sex bias. Among the animal-interaction activities that respondents were asked to report upon, ‘playing’ with animals was not included. Anecdotal observations suggest that children capture and play with small rodents/squirrels, which itself may be an avenue for introduction of MPXV into the human population. Future studies of this nature will benefit from reducing operator-introduced bias stemming from differences in interviewer technique.

Finally, these findings may be applicable to human risk for other viral zoonotic infections with a similar mode of infection. We hope that this work may serve as a guide to map at-risk households, to identify those individuals who may be at risk for either zoonotic or inter-human transmission of MPXV, and to better inform future research.

## Supporting information

S1 Appendix Risk Questionnaire, 2011 version. Risk Questionnaire administered in 2011/2012 (translated from French).
(PDF)

S2 Appendix Risk Questionnaire, 2013 version. Risk Questionnaire administered in 2013 (translated from French).
(PDF)

S3 Appendix Raw data from Risk Questionnaires. Unanalyzed data from all questionnaires.
(XLSX)
