# Supplementary material for: Presumptive risk factors for monkeypox in rural communities in the Democratic Republic of the Congo
Source: PLoS One. 2017 Feb 13;12(2):e0168664. doi: 10.1371/journal.pone.0168664 (PMC5305065; doi:10.1371/journal.pone.0168664)
Supplement: S1 Appendix — Risk Questionnaire administered in 2011/2012 (translated from French). (PDF) [file pone.0168664.s001.pdf]

Household survey on risk factors for transmission of zoonotic diseases with emphasis on monkeypox (DRC 2011/2012)

Date of interview
Number of interview
Educator conducting the interview
Educator recording responses
Department of
Village
Sex
Age
Occupation
Duration in the village

1. Where does the water you and your family drink come from
    a. Spring
    b. River (riviere)
    c. Pond water
    d. River (fleuve)
    e. Wells
    f. Rainwater
    g. Multiple sources
    h. Other (explain)
2. What types and how many pets or wild animals do you have in your household?

**Species (Y/N)**	**amount**	**Species (Y/N)**	**amount**
Monkey		Dog	
Pork		Sheep	
Goat		Cat	
Bovine		Pigeons	
Duck		Guinea pig	
poultry		other	

3. Animals water source
    a. Spring
    b. River (riviere)
    c. Pond of water
    d. Fiver (fleuve)
    e. Well
    f. Rainwater
    g. Many
    h. Other
4. What animals have you hunted, captured, or killed in the last month

Monkey	Turtle	Ecueil
Fish	Pangolin	Bonobo

Porcupine	Pig	Leopard
Bushpig	Rodent	Genet
Blue duiker	Elephant	Muskdeer
Goat	Poultry	Crocodile
Frank black duiker	Peters duiker	Mongoose
Snake	Buiker dorsalis	Duiker syvicultor
other	I don't hunt animals	

5. Who is responsible for preparing this meat
    a. Women
    b. Old women
    c. Men
    d. Young men
    e. Young women
    f. Head of household
    g. Wife
    h. Mother of the child
6. How often do the children go to school
    a. Never
    b. Once per week
    c. Two times per week
    d. Three times per week
    e. 4 times per week
    f. More than 5 times per week
    g. I don't know
    h. No children in the house
7. How many times do you go to church or mosque
    a. Never
    b. Once per week
    c. Two times per week
    d. Three times per week
    e. 4 times per week
    f. More than 5 times per week
    g. I don't know
8. How often do you go into the forest?
    a. Never
    b. Once per week
    c. Two times per week
    d. Three times per week
    e. 4 times per week
    f. More than 5 times per week
    g. I don't know
9. How often do you go to the market?
    a. Never
    b. Once per week
    c. Two times per week

d. Three times per week
e. 4 times per week
f. More than 5 times per week
g. I don't know

10. How many people live in your house
    a. Number of females
    b. Number of males
11. How many people sleep in the same room
    a. Number of females
    b. Number of males
12. What type of animals enter the house when you sleep

Monkey	Turtle	Ecueil
Fish	Pangolin	Bonobo
Porcupine	Pig	Leopard
Bushpig	Rodent	Genet
Blue duiker	Elephant	Muskdeer
Goat	Poultry	Crocodile
Frank black duiker	Peters duiker	Mongoose
Snake	Duiker dorsalis	Duiker syvicultor
other		

13. Do rodents bite children or adults in your house (Y/N/DK)
14. Which rodents do you eat?
    a. Porcupine
    b. Gambian rat
    c. Greater cane rat
    d. Squirrel
    e. Small rat
    f. Other
15. Has a member of your family suffered skin rashes? (Y/N)
16. Was the rash more like photos A/B or C/D
17. Did the person have high fever associated with the rash (Y/N)
    a. Yes before the rash
    b. Yes, during the rash
    c. Yes, after the rash
