# Supplementary material for: Presumptive risk factors for monkeypox in rural communities in the Democratic Republic of the Congo
Source: PLoS One. 2017 Feb 13;12(2):e0168664. doi: 10.1371/journal.pone.0168664 (PMC5305065; doi:10.1371/journal.pone.0168664)
Supplement: S2 Appendix — Risk Questionnaire administered in 2013 (translated from French). (PDF) [file pone.0168664.s002.pdf]

Household survey on risk factors for transmission of zoonotic diseases with emphasis on monkeypox (DRC 2013)

Date of interview____________ Village____________
Number of interview____________ Sex____________
Educator conducting the interview____________ Age____________
Educator recording responses____________ Department of____________
Occupation____________ Duration in the village____________

1. What types and how many pets or wild animals do you have in your household?

Species (Y/N)	Number	Species (Y/N)	Number
Monkey		Dog	
Pork		Sheep	
Goat		Cat	
Bovine		Pigeons	
Duck		Guinea pig	
poultry		other	

_____ I don't own any animals

2. How many people live in your house?

_____ a- females
_____ b- males

3. What material is the floor in your house made of?

_____a- Dirt _____ b- Cement
_____c- Cobblestone _____ d- Other

4. What material are the walls in your house made of?

_____a- Mud _____ b- Wood
_____c- Brick _____ d- Bark
_____e- Other (Explain)________________

5. What material is the roof in your house made of?

_____a- Thatch _____ b- Sheet metal
_____c- Other (Explain)________________

6. What material is the door in your house made of?

_____a- Boards _____ b- Sheet metal
_____c- Wood _____ d- Tiger Palm
_____e- Other (Explain)________________

7. What animals have you encountered in the bush in the last month? Check the appropriate box.

Species	Find	Hunt	Dismember	Eat	Sell	Species	Find	Hunt	Dismember	Eat	Sell
Monkey						Goat					

Allen's swamp monkey						Boar					
cercopithecus Ascanius						Potamochère					
monkey Brazza						Aquatic Chevrotin / doe-pig					
mantled guereza Angola						Duiker					
Bonobo						Duiker was solid black					
Rodent						Peters duiker					
rodent drill						duiker dorsalis					
rodent house						duiker syvicultor					
Cricetomys drill						blue duiker					
grasscutter						Genette					
Squirrel						Leopard					
squirrel wood						Mongoose					
flying squirrel						Poultry					
heliosciure has pink legs						Snake					
Grand squirrel						Tortoise					
Brush-tailed porcupine						Crocodile					
Pangolin						Elephant					
						Other					

_____ I have not hunted animals from the bush
_____ I have not butchered animals from the bush
_____ I have not eaten animals from the bush

8. Who is responsible for preparing this meat?

a. Women
b. Old women
c. Men
d. Young men
e. Young women
f. Head of household
g. Wife
h. Mother of the child

9. How often do the children in your home go to school?

a. Never
b. Once per week
c. Two times per week
d. Three times per week
e. 4 times per week
f. More than 5 times per week
g. I don’t know
h. No children in the house

10. How often do you go to church or mosque?

i. Never
j. Once per week
k. Two times per week

l. Three times per week
m. 4 times per week
n. More than 5 times per week
o. I don't know

11. How often do you go into the forest?

p. Never
q. Once per week
r. Two times per week
s. Three times per week
t. 4 times per week
u. More than 5 times per week
v. I don't know

12. What activities do you do in the forest?

_____a- Hunt _____b- Farm
_____c- Pick up firewood _____d- Picking
_____e- Collecting Water _____f- Other
(Explain)______________________________

13. How often do you go to the market?

a. Never
b. Once per week
c. Two times per week
d. Three times per week
e. 4 times per week
f. More than 5 times per week
g. I don't know

14. What type of animals enter the house when you sleep?

**Species**		**Species**		**Species**	
Monkey		Squirrel		Grasscutter	
Mouse		Pangolin		Snake	
Athrure		Pig		Chicken	
Graphiure (dormouse)		Rat		Goat	
Gambian Rat		Insectes		Other	

_____ Animals do not enter my house

15. Have rodents bitten children or adults in your home?

___________Yes ___________No __________I don't know

16. Have you ever found a dead monkey in the forest?

___________Yes ___________No __________I don't know

If so, what did you do with it?
_____ a- Ate it _____ b- Picked it up
_____ c- Handled it _____ d- Le lassier
